# Retraction: SNHG5 promotes proliferation and induces apoptosis in melanoma by sponging miR-155

**DOI:** 10.1039/d2ra90086f

**Published:** 2022-09-07

**Authors:** Lu Yan, Suihai Wang, Yue Li, Linda Tognetti, Rui Tan, Kang Zeng, Elisa Pianigiani, Xiangbin Mi, Hui Li, Michele Fimiani, Pietro Rubegni

**Affiliations:** Department of Dermatology, Zhujiang Hospital of Southern Medical University Guangzhou 510000 China michelley8051@126.com +86-020-61642833; School of Biotechnology, Southern Medical University Guangzhou 510000 China; Department of Laboratory, Nanfang Hospital, Southern Medical University Guangzhou 510000 China; Department of Medical, Surgical and Neuro Sciences, Section of Dermatology, University of Siena Policlinico Le Scotte Viale Bracci Siena 53100 Italy rubegni@unisi.it +39-0577-40190; Department of Dermatology, Nanfang Hospital, Southern Medical University Guangzhou 510000 China

## Abstract

Retraction of ‘SNHG5 promotes proliferation and induces apoptosis in melanoma by sponging miR-155’ by Lu Yan *et al.*, *RSC Adv.*, 2018, **8**, 6160–6168, https://doi.org/10.1039/C7RA12520H.

The Royal Society of Chemistry hereby wholly retracts this *RSC Advances* article due to concerns with the reliability of the data. Images published in Fig. 2 and 5 of the article have been duplicated in another article.^1^ There are no common authors between the papers.

Rotated and scaled versions of the second and third tumor images in Fig. 5B of this paper are duplicated in the ‘sh-con’ panel in Fig. 6B of ref. 1.

A rotated version of the ‘si-con/SK-MEL-2’ colony formation image in Fig. 2E of this paper is duplicated in Fig. 2D (si-con/MCF-7′ image) of ref. 1. Some additional features can be observed in the image in Fig. 2D of ref. 1, although the majority of the features overlap between the two images.

An inverted version of the ‘si-con/A375’ colony formation image in Fig. 2E of this paper is duplicated in Fig. 2D (‘si-PVT1#2/MDA-MB-231’ image) of ref. 1. Although some features do appear differently in each image, the majority of the features overlap between the two images.

The authors were asked to provide the raw data for this article, but did not respond. Given the significance of the concerns about the validity of the data, and the lack of raw data, the findings presented in this article are not reliable.

The authors were informed but have not responded to any correspondence regarding the retraction.

Signed: Laura Fisher, Executive Editor, *RSC Advances*

Date: 18th August 2022

## Supplementary Material
